# Quality of cochlear implant rehabilitation under COVID-19 conditions

**DOI:** 10.1007/s00106-020-00923-z

**Published:** 2020-10-09

**Authors:** A. Aschendorff, S. Arndt, S. Kröger, T. Wesarg, M. C. Ketterer, P. Kirchem, S. Pixner, F. Hassepaß, R. Beck

**Affiliations:** Department of Oto-Rhino-Laryngology, Universitätsklinikum Freiburg, Killianstr. 5, 79106 Freiburg, Germany

**Keywords:** Speech therapy, Aftercare, Severe acute respiratory syndrome coronavirus 2, Quality assurance, Standard of care

## Abstract

**Background:**

The rehabilitation process following cochlear implant (CI) surgery is carried out in a multimodal therapy according to German national guidelines and includes technical and medical aftercare. In times of the corona pandemic surgery and rehabilitation appointments were cancelled or delayed leading to a more difficult access to auditory rehabilitation. Newly implemented hygiene modalities due to the SARS-CoV‑2 pandemic have changed medical aftercare and the rehabilitation process. The aim of this study was to evaluate the quality of rehabilitation under corona conditions.

**Material and methods:**

An anonymous survey of adult cochlear implant patients was carried out by a non-standardized questionnaire. Demographics were analyzed and the quality of medical aftercare, speech therapy, technical aftercare, psychological support and the hygiene modalities were compared to previous rehabilitation stays.

**Results:**

In total 109 patients completed the questionnaire. The quality of rehabilitation and individual therapy were rated as qualitatively similar or improved. The threat of the pandemic and fear of corona were rated unexpectedly high with 68% and 50%, respectively. The hygiene measures during the rehabilitation stay eased subjective fears at the same time. The majority of patients were annoyed by wearing face masks but visors, protection shields and social distancing were more tolerated.

**Conclusion:**

The implementation of the new hygiene modalities within the therapeutic rehabilitation setting was well-accepted by patients allowing access to auditory rehabilitation. A successful rehabilitation should ensure a fear-free environment by adhering to the necessary hygiene modalities.

**Electronic supplementary material:**

The online version of this article (10.1007/s00106-020-00923-z) includes the study questionnaire. Article and supplementary material are available at www.springermedizin.de. Please enter the title of the article in the search field, the supplementary material can be found under “Ergänzende Inhalte”.

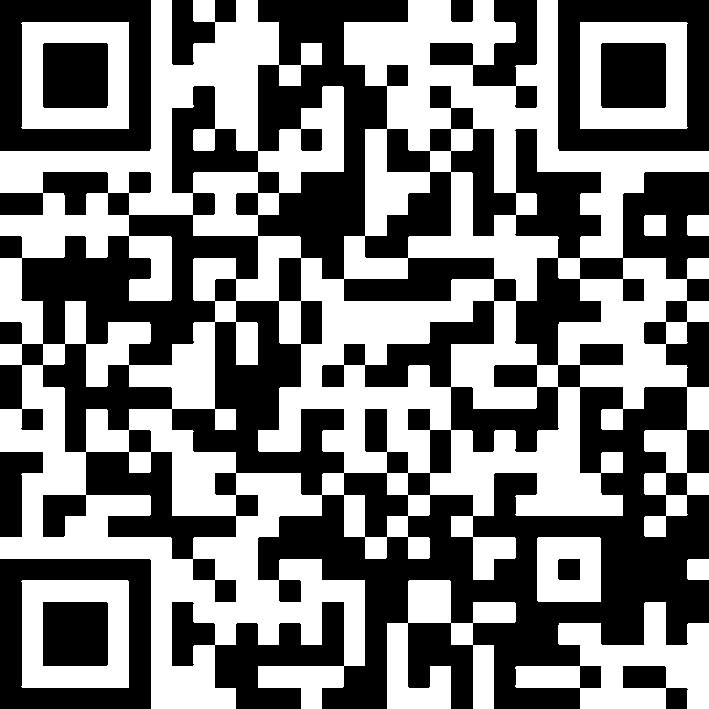

## Background

The coronavirus pandemic has caused massive changes in working procedures in hospitals, general practices, and in rehabilitation clinics. With the start of the so-called lockdown, outpatient services were reduced, operations cancelled or postponed, and rehabilitation programs were no longer conducted or only to a very limited extent after March 17, 2020. Curtailing of elective programs set intensive care capacities free to enable treatment of COVID-19 patients under special hygiene measures. It is estimated that up to 38.9% of surgical interventions in malignant diseases and up to 81.5% in non-malignant diseases were cancelled and/or postponed in the ear–nose–throat (ENT) area [1]. Disruptions to such an extent are of considerable importance both for patients and the health system. Even with operative capacities subsequently increased by 20%, it may take approximately 40 weeks to catch up with this OP backlog [1]. The effects on hearing-impaired patients in particular are not known. However, in the majority of clinics, cochlear implant (CI) surgeries were cancelled or postponed. An analogous assumption can be made for rehabilitation after CI, since at least the follow-up therapy was completely stopped for ca. 2 months. This means that access to auditory rehabilitation was delayed for hearing-impaired patients.

Two months later, with the step-wise relaxing of hygiene measures, surgical capacities were reactivated and the rehabilitation clinics recommenced their work, although to a limited extent. In principle, special hygiene measures, social distancing, and obligatory masks were enforced, along with limited visiting rules and the resultant numerical reduction in capacities.

This situation was found to be a special challenge in rehabilitation after CI. According to the guidelines, rehabilitation is an integral component in CI care [7]. The interdisciplinary treatment comprises: medical care, technical controls, step-wise optimization of the CI processor settings, intensive hearing–speech therapy, multidisciplinary diagnostics (speech and language therapy, phoniatric, pedagogic, and psychological), audiometry (threshold and speech in quiet and in noise), consultation with the patient and their social environment, psychological support, additional training in using the CI system (care, maintenance, malfunction recognition) and in the use of supplementary equipment, documentation and evaluation of results as part of the weekly team conferences, and consultation with social services on the rights of disabled persons and occupational integration. All of this is done to enable inclusion and participation in accordance with the WHO International Classification of Functioning, Disability and Health.

The Q‑Reha-certified CI rehabilitation is conducted for adults at the Implant Centre Freiburg (ICF) on 20 rehabilitation days as an interval rehabilitation. The 5‑day basic therapy is followed by 2‑ to 3‑day rehabilitation stays over the course of 24 months. Due to the coronavirus pandemic, rehabilitation was re-started in early May 2020 with a reduced number of patients. The patients and the persons accompanying them underwent medical examination on admission, including temperature measurement, and were given information on hygiene measures. The usual scheduling plan was modified according to hygiene rules, since group therapies or discussions were not possible. Technical fittings, logopedic diagnostics and therapy, psychological sessions, music therapy, and consultations required the use of mouth–nose masks, face shields, spit barriers, and maintaining the required distance. Meals were served in the cafeteria to reduced numbers and in shifts. Owing to the reduced number of patients to approximately 50% compared with pre-coronavirus times, the patients had a lower chance of contact for personal interaction.

This present study is intended to examine the extent to which the current changes resulting from the application of the required hygiene measures, social distancing rules, and obligatory masks influence the course and the subjective success of CI rehabilitation in comparison with the pre-coronavirus situation. Moreover, the question arises of how CI patients rate the pandemic. To date, there are no studies on rehabilitation after CI under COVID-19 conditions.

## Material and methods

We conducted an anonymous patient survey in the period from May 13, 2020 to June 25, 2020. Adult patients who were attending rehabilitation after CI for the second or multiple times at the ICF were included.

All patients planned for in-patient rehabilitation were contacted by telephone 3–5 days before admission and asked about the state of their health and possible contact with SARS-COV-2-infected persons. An anonymous evaluation was made of the number of patients who wanted to cancel or postpone their appointment.

The hygiene measures were determined in close cooperation and consultation with the Institute for Infection Prevention and Hospital Epidemiology. Social distancing and mouth–nose masks (MNM) had priority. Face shields and spit barriers were used in situations in which distancing or MNM were not possible for medical or therapeutic reasons. Attention was paid to the disinfection of hands according to hospital-wide rules.

The patients who came for rehabilitation were informed about the study during the admission examination and were given written patient information and the questionnaire (Electronic supplementary material online). The latter was handed in anonymously at the end of the 2–3-day stay.

The non-standardized questionnaire comprised 44 questions in the medical, psychological, therapeutic, and technical areas. The survey addressed the qualitative and quantitative comparison of rehabilitation before and under coronavirus conditions. Anxiety regarding the coronavirus pandemic, the quality of the rehabilitation, and the attainment of goals under pandemic conditions were queried. The use of the various hygiene measures—MNM, face shields, spit barriers, and distancing—was evaluated. The importance of exchange among rehabilitation patients was evaluated, as was the reduction in contacts resulting from the hygiene measures. The age group (18–29 years, 30–39 years, etc.), the subjective estimate of belonging to a risk group, rehabilitation experience, inclusion of an accompanying person, and gender were recorded.

The assessment was made descriptively. This study was registered under No. 00021680 with the German Registry of Clinical Studies (Deutsches Register klinischer Studien). With application No. 10020/20 of the Ethics Commission of the University of Freiburg, exemption from obligatory consultation was granted, since only anonymized data were recorded and evaluated.

## Results

A total of 129 questionnaires were issued to rehabilitation patients between May 13, 2020 and June 26, 2020. The response rate was 84.5% (*n* = 109).

Triage results: 48.1% of the adult patients (*n* = 120 of the planned 249 Reha patients) cancelled the appointment during the pre-evaluation by telephone. The distribution of cancellations remained constant over calendar weeks 17–26.

**Fig. 1:**
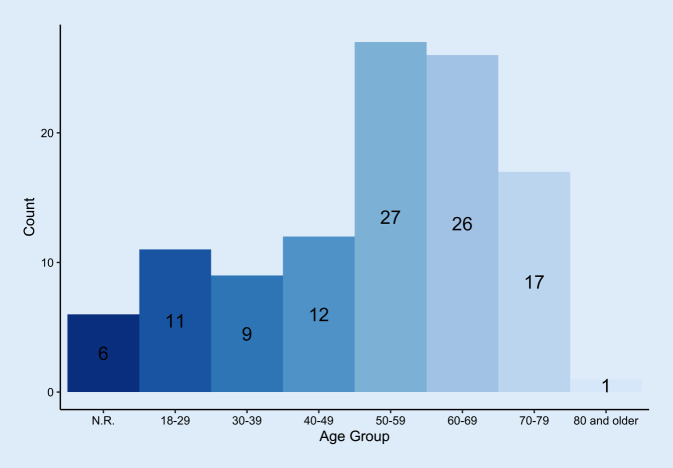
Age distribution of the participants (*n* = 109) in years. *N.R.* no response

**Fig. 2:**
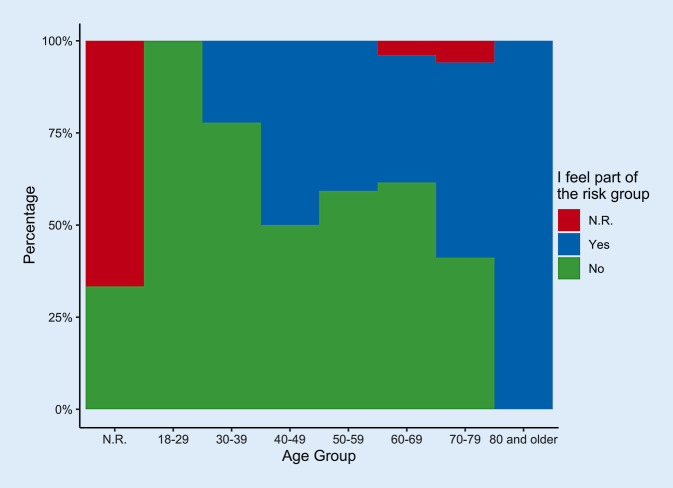
Subjective assessment whether a patient sees him-/herself belonging to a risk group.* N.R.* no response

The age distribution of the patients (*n* = 60 men, *n* = 43 women, *n* = 6 unknown) is shown in Fig. 1. In all, 47.6% of the patients were accompanied by someone. Overall, 63.1% felt they belonged to a risk group. In the various age groups (18–29 years, 30–39 years, etc.), the feeling of belonging to a risk group ranged from 0 to 100%: Young patients did not see themselves belonging to the risk group while 100% of the very old patients (80+ years) felt they belonged to the risk group (Fig. 2). Up to 40% of older patients (70–79 years) did not feel they belonged to the risk group.

The majority of the patients rated the admissions examination as positive: The admitting doctor was sympathetic (86.1%), information on hygiene measures was given (89.8%), and MNM were provided (95.3%).

An initial psychological session is regularly conducted only during basic therapy. During the rehabilitation stay, 18.3% (*n* = 19) of the patients wished for and received an additional session. The majority did not want to broach the issue of coronavirus (15 of 19 patients). Overall, suggestions for everyday problems could be made (13 of 15 patients), the patients felt relieved (11 of 18 patients), and connections could be clarified (14 of 18 patients).

Speech and language therapy as well as technical adjustments of the speech processors were made for all rehabilitation patients and were rated as of constant quality or even better (speech therapy 94.3%, technical aspects 97.1%) compared with pre-coronavirus times. Music therapy was rated as unchanged in quality, but only 44.3% of the patients received this therapy due to the limitation of group therapies.

While 86.9% of the rehabilitation patients rated the intensity of the therapies compared with the pre-coronavirus period as just right, 11.2% found the intensity too low. Overall, 89.6% of the patients could achieve or nearly achieve their therapy goals, while 10.4% could not do so, or only to a limited degree.

The evaluation of whether the ICF offers the proper therapy for each problem was rated positive by 82.4% of the respondents; only three patients (2.5%) were of the opinion that the ICF does not offer the proper therapies.

**Fig. 3:**
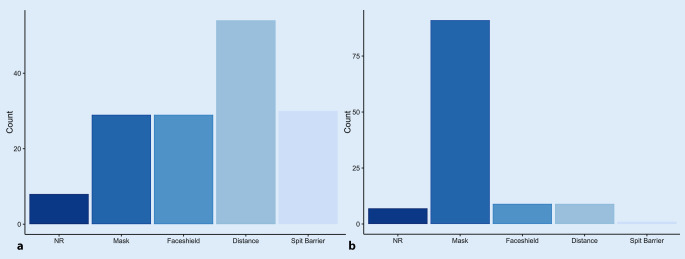
Evaluation of the protective equipment as least (**a**) and most annoying (**b**), including multiple answers. *NR* no response

The use of MNM was rated as annoying by the majority (65.1%), and 48% of the patients considered treatment more difficult due to the masks. Nearly half of the patients (43.6%) considered their own personal mask (MNM) and 25.5% the masks of the therapists annoying; 30.9% of the patients found both irritating (their own mask and the mask of the therapist). The rating of the individual protective measures showed that social distancing was considered the least annoying compared with the face shields, spit barriers, and MNM. The MNM was rated as the most detrimental to therapy (Fig. 3).

The majority of the rehabilitation patients (55.3%) considered conversations with other rehabilitation patients important or very important. The required protective measures negatively affected these conversations to a moderate or high degree for 50% of the patients.

**Fig. 4:**
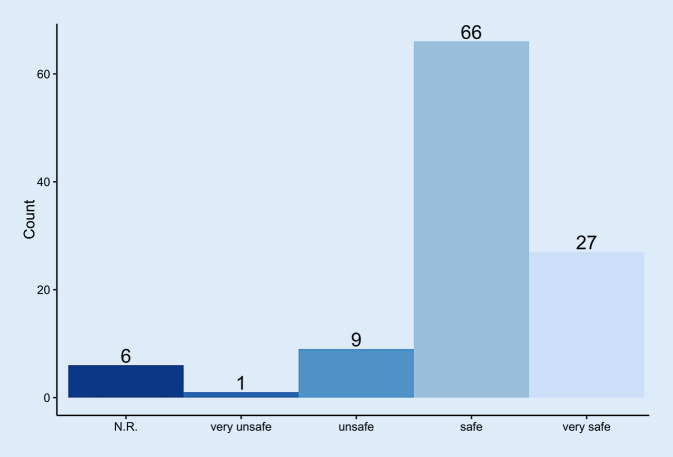
Effect of the hygiene concept expressed as perceived safety in regard to the coronavirus pandemic. *N.R.* no response

For a large majority of the rehabilitation patients (89.3%, Fig. 4), the enforced hygiene measures increased the feeling of security, whereby 68% considered the pandemic as dangerous and 9% as harmless.

The participating rehabilitation patients reported fear of coronavirus in general in 50% of cases, equally distributed over all age groups. In the rehabilitation clinic, i.e., during the current stay, this feeling ebbed, so that 81.5% felt little or even no fear, which correlated with the answer “no fear of coronavirus at the moment” by 75%; a subjective increase in the feeling of fear in the clinic was reported by 18.4% and “at the moment” by 25%.

## Discussion

The necessity of postoperative rehabilitation after CI surgery is well accepted in Germany and described in national guidelines [7, 8]. There are various outpatient and inpatient concepts that use interval or block rehabilitation [12, 17]. Common to all of these is the interdisciplinary approach to therapy to achieve the best possible participation and inclusion as defined by the WHO. Under pandemic conditions it must be assumed that the access to auditory rehabilitation for hearing-impaired people is more difficult. This is due to the reduction in the number of operations as well as to the discontinuation of rehabilitation or limitations of technical support.

This study describes the subjective effects on the rehabilitation patients arising from the required measures after rehabilitation was possible again. The return rate of 84.5% of the anonymous questionnaires can be rated as extremely positive and probably expresses, on the one hand, the great interest of the rehabilitation patients in the survey, but also the desire to submit an opinion of the rehabilitation process. Thanks to the high return rate, the survey could be completed within a short time, so that an adaptation effect to the required measures is not likely. The constant rate of cancelled rehabilitations during the telephone pre-evaluation over time also makes an adaptation effect unlikely.

The explanations of the hygiene measures by the doctor were felt to be positive and guaranteed the rehabilitation stay. However, this occurred with a reduced number of patients and means that there is still a patient backlog that is not decreasing. Another problem is the cancellation by patients who postpone their rehabilitation stay “until later” because they are worried about infection during the stay or during the trip (e.g., train travel). This creates a further patient backlog. Moreover, shifts within an interval rehabilitation, whether as in- or outpatient, always bring a risk that the interval between the individual therapies and adjustments will be too long, so that the full potential will not be achieved or there will be programming that needs improvement, resulting in limited speech comprehension. For deaf and hearing-impaired patients, it also applies that chronic disease does not take a “Corona break” [13].

The use of hygiene measures made the accustomed rehabilitation more difficult. The MNM was considered to be the most annoying for patients as well as the most disruptive for treatment. Studies by Saile and Gregori in 2020 [6] showed a muting effect of MNM of up to 10 dB. For hearing-impaired patients, who may also be dependent on lip-reading, this is a massive limitation to communication, which was reflected in the survey results. The use of transparent MNM or face shields may ameliorate this effect, whereby in our study, face shields as well as spit barriers were in fact rated better by comparison. This must be taken into account for the care of hearing-impaired patients in practice. At least a face shield should be immediately available to enable meaningful communication between the doctor or therapist and the patient. Muting due to the MNM should be taken into account in programming. Muting and frequency-specific programs for the mask situation and programs for no-mask situations, for example, in the private sphere, are plausible. The use and problems of MNM are addressed in the websites of the Deutscher Schwerhörigenbund e. V. and the Landesverband der Gehörlosen Baden-Württemberg (self-help groups for the hearing-impaired and deaf community), among others [9, 10].

It is interesting that the rehabilitation patients definitely noticed the limitations due to hygiene measures, but rated the quality of rehabilitation as unchanged or even improved. This applies to all areas of rehabilitation, such as logopedics, technical aspects, and music therapy. The altered setting with MNM, face shields, spit barriers etc. had no negative effect on the rehabilitation success and achieving of goals. This can be interpreted, on the one hand, as an expression of acceptance of the required measures by the patients, but also as the successful and empathetic implementation of the measures by the doctors, therapists, and technicians.

Fear of the coronavirus pandemic in the general public has been cited at ca. 30% since early May 2020 [2]. In the present study, however, the adult rehabilitation patients rated the pandemic as dangerous in 68% of cases, with 50% reporting being afraid of the coronavirus. This gives a clear indication that hearing-impaired and CI patients rate the pandemic differently than the general public do. This may be due to real and felt limitations of communication, freedom of movement, and access to hearing rehabilitation and it correlates with the results of the Mannheim Corona Study of People with Reduced Health (Mannheimer Corona-Studie für Menschen mit eingeschränkter Gesundheit; [15]). It is interesting that the majority of participants reported a reduction in the feeling of fear of the coronavirus during rehabilitation. This can be interpreted as relief in the clinic setting and probably reflects the positive influence of the required hygiene measures and changed medical advice on admission. This finding is also associated with the lower proportion of patients (only 18.3%) who wanted psychological support and were practically (78.9%) not interested in a discussion of the coronavirus.

We only surveyed patients who were at the ICF for therapy and who did not cancel or postpone their stay because of concerns about the pandemic, which is a limitation of this study. This limitation also means that hearing-impaired or CI patients may possibly experience limitations and anxiety due to coronavirus to a much higher extent than the normal population. This is particularly important as it must be postulated that especially anxious patients are more likely to cancel the rehabilitation appointments. For further studies, structured interviews with this patient group are important so as to optimize dealing with the anxieties of hearing-impaired patients in times of pandemics.

Another limitation of this study is the use of a nonstandardized questionnaire. This was applied due to the completely new situation of a worldwide pandemic and the actual relevance of the rehabilitation subject and because of non-existing validated questionnaires.

The results of our study are a confirmation of the hygiene measures implemented at the ICF. The hygiene concept does not only affect the patients but needs to demonstrate the care of management for the therapists and ensure safety for the caregivers, creating a cool-headed and quiet working atmosphere. The team at the ICF put every effort into creating such a workspace. Weekly telephone conferences of management including all department heads and colleagues in charge of quality management were very helpful. The topics discussed are made transparent by distributing the minutes to all staff members. Indirectly, all this was evaluated positively by the patients since the risk posed by the coronavirus was generally perceived as high, but at the ICF as low.

## Outlook

The current pandemic situation leads to limitations for hearing-impaired patients. Access to auditory rehabilitation appears more difficult. Despite all relaxation to date, a high percentage of these patients cancel their rehabilitation appointments. This may mean that patients use their speech processors with inadequate settings over longer periods and are thus not able to achieve their full hearing potential; this is contrary to the goal of inclusion.

The use of electronic media for rehabilitation is common in Germany in individual cases to support rehabilitation (learning apps etc.), but it could provide another form of therapy in the future or supplement existing concepts. There are reports now on remote mapping [14], but this is seldom used in the German-speaking region. In the Anglo-American area, in which rehabilitation as we know it does not exist [3], positive effects of online auditory training are described [4, 5, 16]. However, the question arises of whether these forms of training alone (mapping plus auditory training) meet the term “rehabilitation” according to the WHO [12], and financing such models has not been clarified for the German-speaking region thus far. Basically, the increased use of electronic media, analogous to the home office, in combination with classic rehabilitation on site is plausible in order to increase the hearing potential of patients [11]. Further evaluations of online rehabilitation are needed, however, to test the efficiency and effectiveness, but also to disclose its limitations. The contact between the rehabilitation patients should not be underestimated in guaranteeing and optimizing rehabilitation. The majority of the patients surveyed considered these contacts important and complained of the limitation of these social contacts due to the required hygiene measures and the reduced number of patients.

The “Krankenhausentlastungsgesetz” (German national legislation) supports hospitals and rehabilitation clinics financially during the coronavirus pandemic. This support will not be sufficient to balance the financial losses, according to our own experience. The currently necessary reduction in patient numbers and the changes within the rehabilitation process according to the newly implemented hygiene measures (e.g., no group sessions or group sessions with reduced number of patients, time-consuming preparation of therapy rooms) will increase the length of waiting lists further. This will make the rehabilitation process more expensive and should be considered in future negotiations with health insurance providers.

## Practical conclusion

- A successful, relatively fear-free rehabilitation under COVID-19 conditions is possible.
- The patients can effectively adapt to the situation, even if hygiene measures, especially the mouth–nose mask (MNM), are considered annoying by the majority.
- The use of face shields or transparent MNM should be self-evident for facilitated communication.
- The task for all those involved in rehabilitation is to create a safe environment for the optimal auditory rehabilitation under the best-possible protection for patients and coworkers, even under pandemic conditions.

## Caption Electronic Supplementary Material

Study questionnaire
